# Minimal Mesoscale Model for Protein-Mediated Vesiculation in Clathrin-Dependent Endocytosis

**DOI:** 10.1371/journal.pcbi.1000926

**Published:** 2010-09-09

**Authors:** Neeraj J. Agrawal, Jonathan Nukpezah, Ravi Radhakrishnan

**Affiliations:** 1 Department of Chemical and Biomolecular Engineering, University of Pennsylvania, Philadelphia, Pennsylvania, United States of America; 2 Department of Bioengineering, University of Pennsylvania, Philadelphia, Pennsylvania, United States of America; 3 Department of Biochemistry and Biophysics, University of Pennsylvania, Philadelphia, Pennsylvania, United States of America; University of Illinois at Urbana-Champaign, United States of America

## Abstract

In eukaryotic cells, the internalization of extracellular cargo via the endocytic machinery is an important regulatory process required for many essential cellular functions. The role of cooperative protein-protein and protein-membrane interactions in the ubiquitous endocytic pathway in mammalian cells, namely the clathrin-dependent endocytosis, remains unresolved. We employ the Helfrich membrane Hamiltonian together with surface evolution methodology to address how the shapes and energetics of vesicular-bud formation in a planar membrane are stabilized by presence of the clathrin-coat assembly. Our results identify a unique dual role for the tubulating protein epsin: multiple epsins localized spatially and orientationally collectively play the role of a curvature inducing capsid; in addition, epsin serves the role of an adapter in binding the clathrin coat to the membrane. Our results also suggest an important role for the clathrin lattice, namely in the spatial- and orientational-templating of epsins. We suggest that there exists a critical size of the coat above which a vesicular bud with a constricted neck resembling a mature vesicle is stabilized. Based on the observed strong dependence of the vesicle diameter on the bending rigidity, we suggest that the variability in bending stiffness due to variations in membrane composition with cell type can explain the experimentally observed variability on the size of clathrin-coated vesicles, which typically range 50–100 nm. Our model also provides estimates for the number of epsins involved in stabilizing a coated vesicle, and without any direct fitting reproduces the experimentally observed shapes of vesicular intermediates as well as their probability distributions quantitatively, in wildtype as well as CLAP IgG injected neuronal cell experiments. We have presented a minimal mesoscale model which quantitatively explains several experimental observations on the process of vesicle nucleation induced by the clathrin-coated assembly prior to vesicle scission in clathrin dependent endocytosis.

## Author Summary

Cell membranes and membrane-based organelles actively mediate several intracellular signaling and trafficking decisions. A growing number of applications rely on cooperative interactions between molecular assemblies and membranes. Yet, the studies of membrane-based and membrane-mediated signaling are not considered core aspects of systems biology. While a coherent and complete description of cell membrane-mediated signaling is not always possible by experimental methods, multiscale modeling and simulation approaches can provide valuable insights at microscopic and mesoscopic scales. Here, we present a quantitative model for describing how cell-membrane topologies are actively mediated and manipulated by intracellular protein assemblies. Specifically, the model describes a crucial step in the intracellular endocytic trafficking mechanisms, i.e., active transport mechanisms mediated through budding of the cell membrane orchestrated by protein-interaction networks. The proposed theory and modeling approach is expected to create avenues for many novel applications in systems biology, pharmacology, and nanobiotechnology. The particular application to endocytosis explored here can help discern pathological cellular trafficking fates of receptors implicated in a variety of biomedical conditions such as cancer, as well as impact the technology of targeted drug delivery in nanomedicine.

## Introduction

The cellular process of endocytosis is important in the biological regulation of trafficking in cells, as well as impacts the technology of targeted drug delivery in nanomedicine [1], [2], [3], [4], [5], [6], [7]. In eukaryotic cells, the internalization of extracellular cargo via the endocytic machinery is an important regulatory process required for many essential cellular functions, including nutrient uptake and cell-cell communication. Several experimental [8] as well as theoretical [9], [10], [11] treatments have addressed mechanisms in endocytosis, yet the role of cooperative protein-protein and protein-membrane interactions in the ubiquitous endocytic pathway in mammalian cells, namely clathrin-dependent endocytosis (CDE), remains unresolved. A sequence of molecular events in CDE is responsible for the recruitment of adaptor protein 2 (AP-2), accessory proteins such as epsin, AP180, Eps15, Dynamin, etc., and the scaffolding protein clathrin to the plasma membrane [8]. The accessory proteins such as epsin are implicated in membrane bending [12]. Polymerization of clathrin triskelia in the presence of adaptor proteins such as AP-2 results in the clathrin coat formation, and tubulating proteins such as epsin interact with both the clathrin coat as well as the bilayer [13] to stabilize a clathrin-coated budding vesicle. The involvement of dynamin is believed to be in the vesicle scission step [8]. Even though actin is believed to play an important role in the endocytosis process in *S. cerevisiae* (yeast), in mammalian cells, actin repression, at best, has a small effect on endocytosis [14].

We focus on the energetic stabilization of a budding vesicle induced by the clathrin-coat assembly. Recent work [15] demonstrates that the membrane invagination only begins in the presence of a growing clathrin coat [16]. Experiments performed by down-regulating AP-2 expression [17], [18] as well as those involving the inhibition of epsin [19] either significantly decrease the number of clathrin-coated pits or alter the distribution of coated-intermediates involved in the vesicle-bud formation. Although the CDE in mammalian cells remains a complex regulatory process, we believe that a critical and self-consistent set of experiments is now emerging which warrants the formulation of physically-based models to quantitatively describe the bioenergetics of protein-induced vesicle formation in CDE [20].

**Figure 1:**
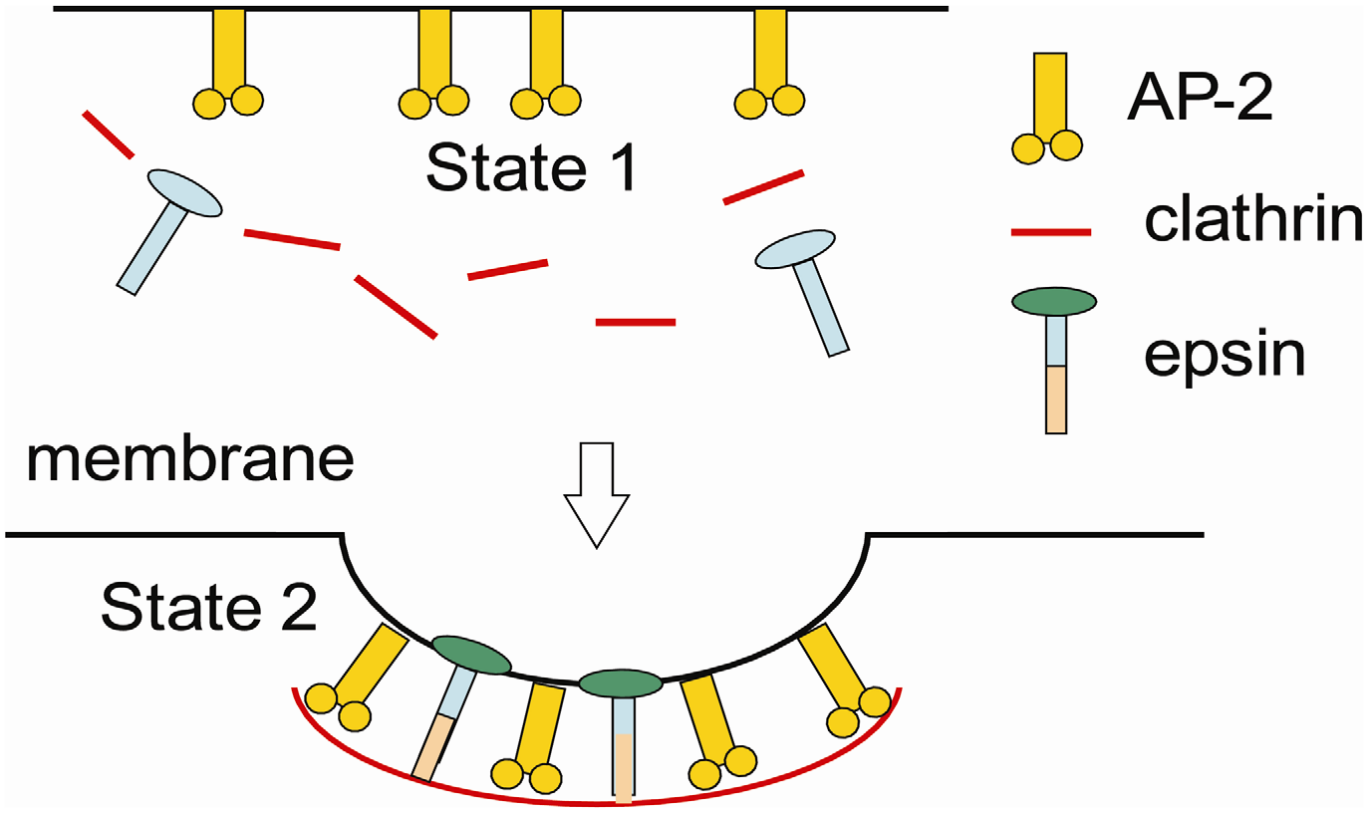
Reaction scheme for the clathrin coated vesicle formation. The free energy of state 2 relative to state 1 is described by E_t_.

Even though models directly addressing CDE in the experimental (cellular) context have not been proposed, Oster et al. have addressed yeast endocytosis driven by actin [9], [21]. Moreover, Kohyama et al. [22] have shown that model two component membranes bud in response to induced spontaneous curvature or the line tension between the two components of the membrane and Frese et al. have investigated the effect of protein shape and crowding on domain formation and curvature in biological membranes [23]. A recent mini-review examining the current experimental trend by Lundmark and Carlsson on driving membrane curvature in clathrin-dependent and clathrin-independent endocytosis is also available [24]. We formulate a minimal model, by restricting our focus to three proteins in the clathrin-coat assembly (Fig. 1): clathrin, epsin and AP-2, and their role in the stabilization of a budding vesicle on the cell membrane. Mammalian cells have a diverse set of proteins which often serve as surrogates and participate in compensatory mechanisms. In this regard, our choice for the ingredients for the minimal model represents roles for the scaffolding proteins (clathrin), curvature inducing proteins (epsin) and the adaptor proteins (AP-2). Recent experiments [15], [25] have reported characteristics of nucleation and growth of clathrin coat: the initiation was observed to occur randomly, but only within subdomains devoid of cytoskeletal elements. In BSC1 cell lines, such domains appear to be 400 nm in diameter surrounded by a rim of a 200 nm “dead zone”. Notably, the nucleation of clathrin coats was observed only in the 400 nm region [25] with the following salient properties: (a) in the growth phase, the addition of clathrin proceeds at a steady rate of about one triskelion every 2 s, (6s-old coats have 10–20 clathrins). (b) Two fates are possible for a growing coat; they either transform into a vesicle (in 32 s the structure resembles a coated vesicle, 50–100 nm in diameter depending on cell type), or they abort containing about 10–40 triskelia, which suggests that the coat sizes are bounded. While we do not consider the process of nucleation and growth of clathrin, based on the above observations, we study the process of one maturing vesicle in the presence of an assembled clathrin coat of a finite size in a membrane patch free of cytoskeletal elements and subject to a pinned boundary condition at the patch boundary. For our model cell membrane patch not fortified by cytoskeleton, we employ a typical value of bending rigidity of our κ = 20k_B_T derived from literature [26], [27]; (we also explore the effect of varying κ). In this respect, we describe a mean-field model which characterizes the membrane patch as a homogeneous phase with effective (bulk-like) properties. Our model is also mean-field in the sense that it applies to just one vesicular intermediate and the effect of neighboring coats is not included. As noted earlier, our model does not account for the mechanism of clathrin coat nucleation or that of vesicle scission.

Clathrin triskelia and AP-2 (in a ratio of 1∶1) polymerize to form a coat [28] and the stabilizing interactions in the clathrin coat assembly can be quantified using the free energy of the polymerization process. Based on *in vitro* equilibrium data of clathrin cage formation, Nossal [29] estimated the energetics of a fully-closed clathrin/AP-2 basket relative to a dissolved coat to be ≈−20 k_B_T. The inclusion of epsin in the clathrin-coat accounts for −23 k_B_T of energy per bound epsin: the ENTH domain of epsin binds to the PtdIns(4,5)P_2_ (or PIP2) lipid head groups on the membrane with a binding energy of −14 k_B_T per bound epsin [12] and the CLAP domain of epsin interacts with clathrin/AP-2 with an energy of −9 k_B_T [30]. The ENTH interactions with the membrane require the presence of PIP2, which constitutes about 1% of the total phospholipids on the cell membrane [31]. To produce a coated vesicle d = 50 nm diameter, (based on the empirical scaling relationship, the number of triskelia involved ∼0.031d^7/4^ is 29 [29]), the area of the clathrin coat required is πd^2^ = 7850 nm^2^. Considering the area per lipid head-group to be 0.65 nm^2^, the number of PIP2 molecules in the membrane spanning the area of the coat is 1% of (7850/0.65) = 185. Hence, we note that the ratio of ENTH binding sites (which correspond to the PIP2 on membrane) to the CLAP binding sites (which correspond to the triskelia) is 185/29≈6, and hence as the clathrin coat grows, we expect sufficient number of the corresponding PIP2 binding sites to be present for the ENTH domain of epsin to bind. For this reason, we are justified in not explicitly considering PIP2 as a necessary/limiting species in our minimal model.

## Methods

**Figure 2:**
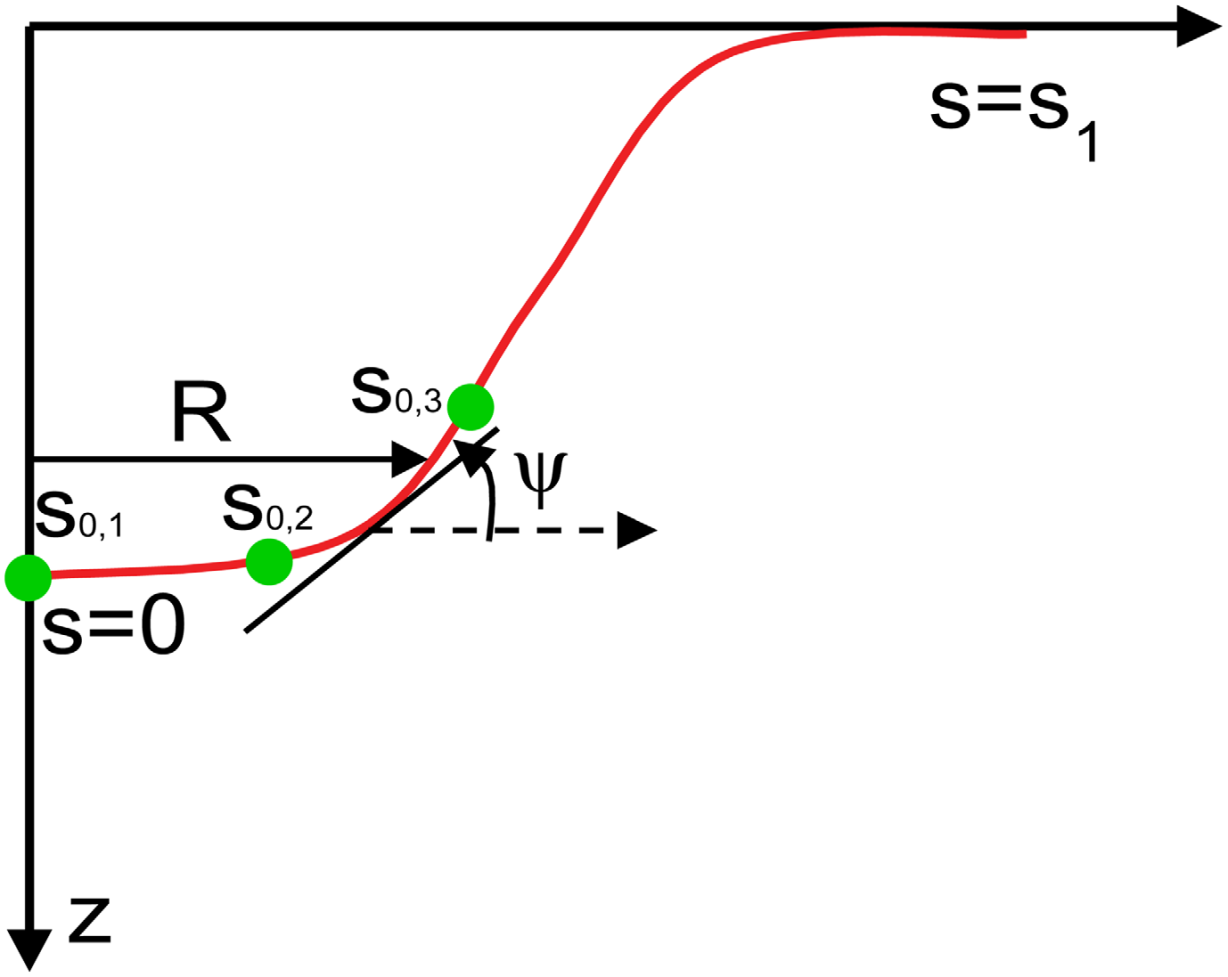
A schematic of the membrane profile explaining the variables in the surface evolution methodology. The full membrane profile is obtained by rotating the curve by 2π about the z-axis.

Field-theoretic approaches are popular for studying energetic and entropic contributions in continuum field-based mesoscale models [32], [33] and several successful applications of such mesoscale models for gaining mechanistic insight into cell-membrane mediated processes are available [3], [9], [21], [34], [35], [36]. Here, to model membrane response in CDE, we solve the membrane equations in a curvilinear manifold by assuming an underlying axis-symmetry using the surface evolution formalism outlined by Seifert et al. [37]. We derive the equations governing membrane shapes of minimum energy under imposed curvature fields assuming that curvature fields are additive and that protein insertion does not cause spatial heterogeneities in physical properties of membrane such as bending rigidity and interfacial frame tension. Parameterizing the membrane shape by the angle 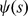, where s is the arc-length along the contour, we obtain 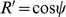 and 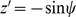, where prime indicates the derivative with respect to arc-length s, (Fig. 2). As described by Safran [38], for topologically invariant membrane shape transformations, the contribution of the Gaussian curvature term to the membrane deformation energy is a constant. Hence, we describe the membrane energy, E using the Helfrich formulation [39]. By including only one of the two principal curvatures, namely the mean curvature:
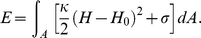
(1)


Here, H is the mean curvature of the membrane, H_0_ is the imposed (or intrinsic) curvature of the membrane due to curvature-inducing proteins and is a function of arc-length s, 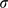 is the membrane interfacial frame tension and A is the total membrane area. We express curvature H and the area element dA in terms of 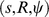. Minimization of this energy functional with respect to 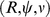 leads to (see Text S1):
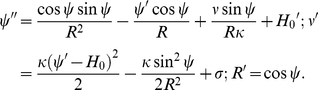
(2)


Here, 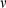 is a Lagrange multiplier introduced to satisfy the constraint 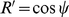 (which defines *R*). We also impose the boundary condition 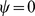 at R = R_0_ (or at s = s_1_) corresponding to the pinning of the membrane by the cytoskeleton at the boundary of the membrane patch. In addition, due to the axis-symmetry, at R = 0, 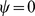. Since the total arc-length s_1_ is not known *a priori*, one additional closure equation is specified, (see Text S1): 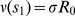. We solve the above system of boundary valued differential equations numerically by the shooting and marching technique [40], (see Text S1), yielding membrane profiles for a specified spontaneous curvature function, and pinned at R = R_0_; in this work, we employ R_0_ = 500 nm. We also compute the curvature deformation energy of the membrane defined by:
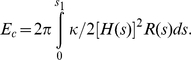
(3)


We present our results for the case when interfacial frame tension σ is zero. Results obtained for non-zero σ (not shown) are found to be similar to the σ = 0 case. We also note that in prior work, we showed that the entropic term |TΔS| at T = 300K is small, i.e. ∼5% of the membrane bending energy for κ = 20 k_B_T [41]. This result justifies the basis for neglecting thermal fluctuations (such an assumption was also employed by Oster et al. for their model for endocytosis in yeast [9]) and is valid except in cases where the vesicle neck region becomes narrow (i.e. same order of magnitude as the bilayer thickness). The situation of a narrow vesicle neck is very pertinent to vesicle scission, where even the continuum treatment of the membrane is subject to approximations and a molecular treatment is necessary as described by Lipowsky et. al, recently [42]. For a given membrane profile, the area of the coat A_a_(s_0_) is computed using the relationship,
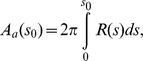
(4)where, the neck-radius R(s_0_) is the radius at s_0_, which marks the coat boundary.

## Results

### Orchestration of a Budding Vesicle in CDE

In our model, the dominant factor contributing to the intrinsic curvature H_0_ in the region where the membrane binds to the clathrin coat is the presence of epsins, bound at the CLAP-binding sites on the coat. In a recent study, [11], we modeled the spontaneous curvature induced by one epsin as a Gaussian function:
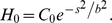
(5)


That is, for the nature of epsin-induced curvature, we have assumed a form that has a spatial decay. Such a choice of spatially-varying intrinsic curvature function is motivated by recent molecular simulations [35], [36], [43], [44]. We have also employed such models in our earlier work [11], [45]. Similarly, for integral membrane proteins, a local curvature model has been proposed by Goulian et al. [46], Oster et al. [47], and Lubensky et al. [48]. Hence there is a bank of such phenomenological curvature models in use in the literature.

In vitro, Ford et al. [12] observed tubulation of vesicles upon addition of epsin; the observed tubule diameter of 20 nm enables us to estimate C_0_ = 0.1 nm^−1^. Using the surface-evolution approach, we calculate the curvature deformation energy of the membrane, E_c_ (defined in Eq. (3)) when a single epsin interacts with the membrane, i.e. through the curvature function in Eq. (5). Since the energy E_c_ is stabilized by the negative interaction energy of the ENTH domain of epsin with the membrane (E_r_), we iteratively determine the value of b in Eq. (5) such that E_c_≈|Er|; using E_r_ = −14k_B_T [12], we obtain b = 8.3 nm for κ = 20 k_B_T.

The periodicity of clathrin lattice, (from cryo-EM studies [49], the average distance between adjacent vertices of the hexagons in the clathrin cage is 18.5 nm), ensures that epsins are templated to maintain both spatial as well as bond-orientational ordering [50]. Hence, within our axis-symmetric membrane model, we translate the patterning of epsins on the clathrin coat to an intrinsic curvature function H_0_ of the form:
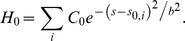
(6)


**Figure 3:**
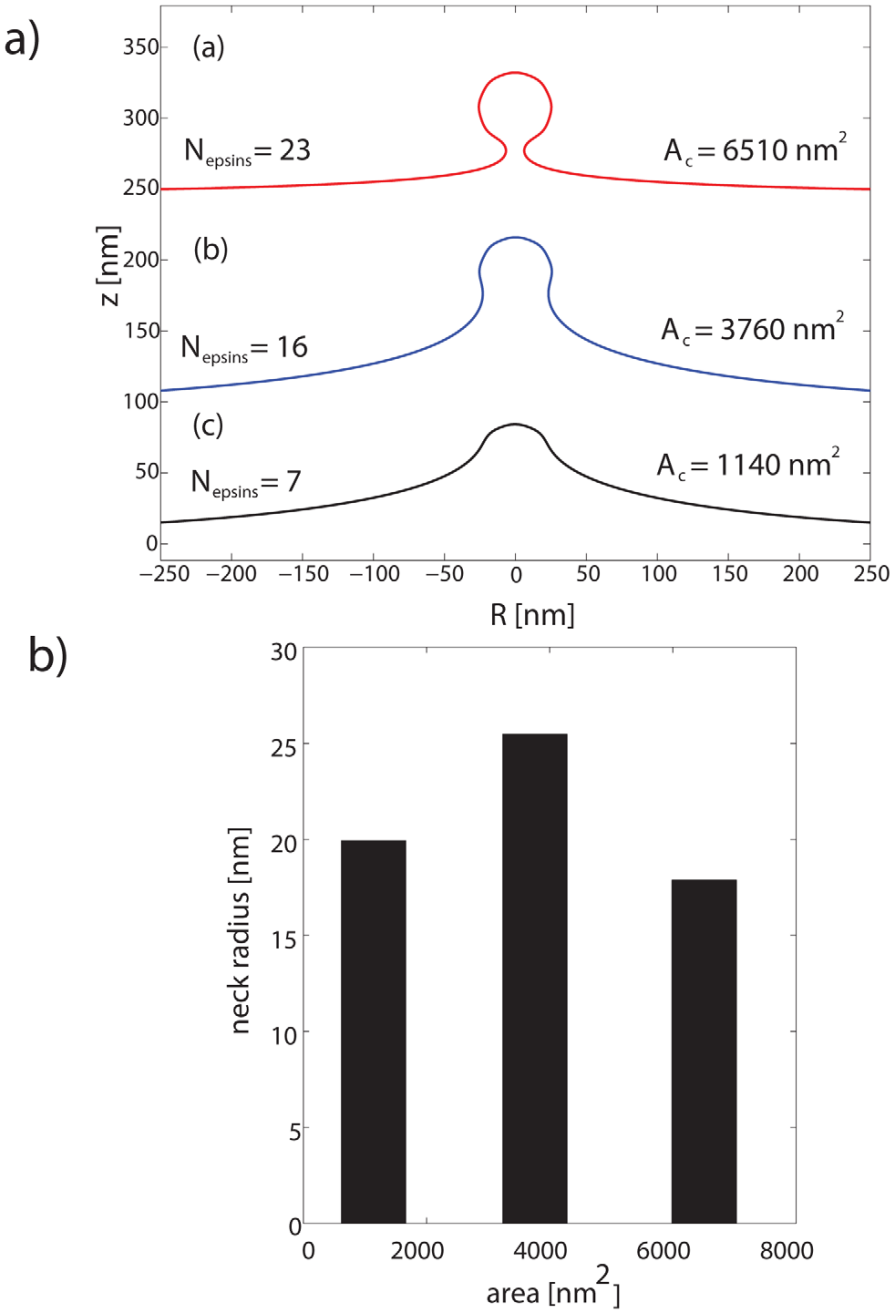
Membrane deformation profiles under curvature fields. (**a**) Three different membrane deformation profiles under the influence of imposed curvature of the epsin shell model for three different coat areas; here κ = 20 k_B_T. For the largest coat area, the membrane shape is reminiscent of a clathrin-coated vesicle. (**b**) Vesicle neck-radius as a function of coat area A_a_.

Here, the index i runs over the number of concentric shells of epsins on the coat separated by a distance of 18.5 nm, the underlying periodicity of the clathrin lattice. Hence, relative to a central epsin bound to the coat at R = 0 and s_0,1_ = 0, successive shells of epsins are located at s_0,2_ = 18.5 nm, s_0,3_ =  37 nm, s_0,4_ = 55.5 nm, etc. until we reach the periphery of the coat of a prescribed extent (or area A_a_); the H_0_ function is depicted in Fig. S5 and the schematic location of the shells is also depicted in Fig. 2. We note that the coat boundary is prescribed by the value of s_0_ for the outermost shell and the neck-radius R(s_0_) is the radius at this value of s_0_, as described earlier. In Fig. 3a, we depict energy minimized membrane deformation profiles for different values of the clathrin coat area A_a_ (defined in Eq. (4)) obtained using the surface evolution method and subject to the epsin curvature fields described by Eq. (6); we find that above a critical value of the coat area, the membrane profile develops overhangs, (also evident from the behavior of the neck-radius in Fig. 3b), which when the coat area A_a_ approaches 6500 nm^2^, transforms to a mature spherical vesicular bud with a narrow neck. We emphasize the generality of this result, i.e., that there exists a critical coat area above which the membrane deformation develops an over-hang and a constricted neck, by confirming this observed trend using a conceptually simplified “capsid model” in which H_0_(s) = 0.08 nm^−1^ if s<s_0_ and H_0_(s) = 0 if s≥s_0_, s_0_ is the length of the clathrin coat, as described in Text S2 and Fig. S1. In Fig. 3a, we estimate the number of epsins, N_epsins_,_i_ in each shell i as:
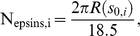
(7)where, R is in nm, and 18.5 (nm) represents the triskelial spacing underlying the clathrin lattice; R(s) is depicted in Fig. S3. The total number of epsins is obtained by summing over the number of shells, which for the mature vesicular bud is estimated to be 23, see (a) in Fig. 3a.

### Effect of Bending Rigidity

**Figure 4:**
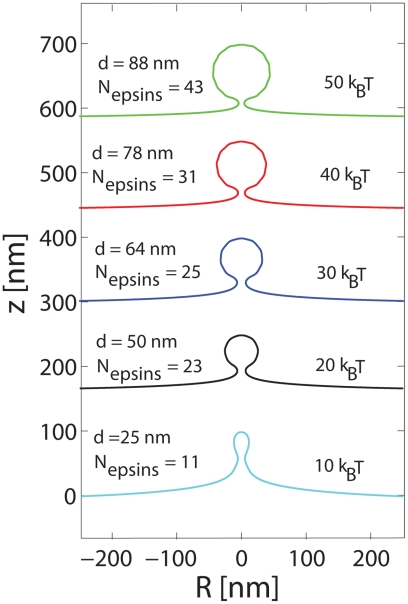
Membrane deformation profiles for mature vesicular buds under the influence of imposed curvature of the epsin shell model for different values of the membrane bending rigidity.

Our results for the epsin shell model assumed a value for the bending rigidity of κ = 20 k_B_T reported in the literature [26], [27]. However, membrane bending rigidity depends upon multiple factors: membrane lipid and protein composition, anchoring of lipid with cytoskeleton, etc. Hence, a broad range of bending rigidity, 10–400 k_B_T has been reported in the literature: in particular, there is consensus that cytoskeleton-free membranes have rigidity in the range of 20 k_B_T and cytoskeleton-fortified membranes can be as stiff as 400 k_B_T. For this reason, it has indeed been postulated that apparent bending rigidity of the membrane depends on the relevant length scale and lies between 20 k_B_T (membrane patches below 100 nm) and 500 k_B_T (membrane patches of 1 µm) [26]. Hence, we have further explored the effect of varying κ in the range κ = 10–50 k_B_T on the mechanism of epsin-induced vesicular bud formation. In Fig. 4, we plot the membrane profiles for a mature vesicle for different values of κ. We note that, in varying κ, we also self-consistently determined the value of b (the range of epsin curvature) as outlined earlier: the dependence of b on membrane bending rigidity is shown in Fig. S4. For each value of κ, we varied the number of shells i in Eq. (6) to solve for the membrane profiles and determined the number of shells necessary for obtaining a mature vesicle; N_epsins_ and the diameter of the vesicular bud, d, were also computed as depicted in Fig. 4. The membrane profiles in Fig. 4 suggest that the epsin-shell model is still viable in orchestrating a mature vesicular bud for different values of membrane bending stiffness. However, we note that there is a strong dependence of the bud diameter on the bending rigidity, which suggests that the variations of in the size of the vesicle in CDE across cell types could be due to changes in the effective bending rigidity of the membrane.

### Energy Considerations in the Stabilization of a Budding Vesicular Intermediate in CDE

The computed deformation energy E_c_ (defined in Eq. (3)) for the capsid model is plotted in Fig. S2 and is seen to increase linearly with increasing coat area, A_a_; we find that the energy E_c_ required to form a mature spherical bud of diameter 50 nm is estimated to be 25κ = 500 k_B_T. The estimate is very close to 8πκ, which is the deformation energy of a spherical vesicle of diameter d for which H_0_ = 4/d (and constant in space). The energy E_c_ required to deform the membrane can be offset by stabilizing interactions between the proteins in the clathrin coat assembly and between the coat proteins and the membrane. As described in the introduction, the free energy of the clathrin-coat assembly, E_a_ was estimated by Nossal [29] to be ≈−20 k_B_T, i.e., |E_c_|≫|E_a_|. This implies that the curvature induction in the presence of a clathrin-coat is energetically unfavorable in the absence of additional stabilizing interactions. Indeed, as reported in cell-experiments [25], not all growing clathrin coats result in vesiculation events and a commitment step possibly accounting for additional stabilizing interactions (E_r_ which includes those interactions that preferentially stabilize state 2 over state 1 in Fig. 1) is necessary. As noted in earlier 1, inclusion of epsin in the clathrin-coat accounts for ε_epsin_ = −23 k_B_T per bound epsin and hence, within our model, we consider E_r_(A_a_) = N_epsins_(A_a_)×ε_epsin_. Thus, for a given extent of the coat characterized by its area A_a_, the total free energy change of the membrane and clathrin-coat assembly in the curved state (state 2, see Fig. 1) relative to the planar state (state 1, see Fig. 1) is given by: E_t_(A_a_) = E_c_(A_a_)+E_a_(A_a_)+E_r_(A_a_).

**Figure 5:**
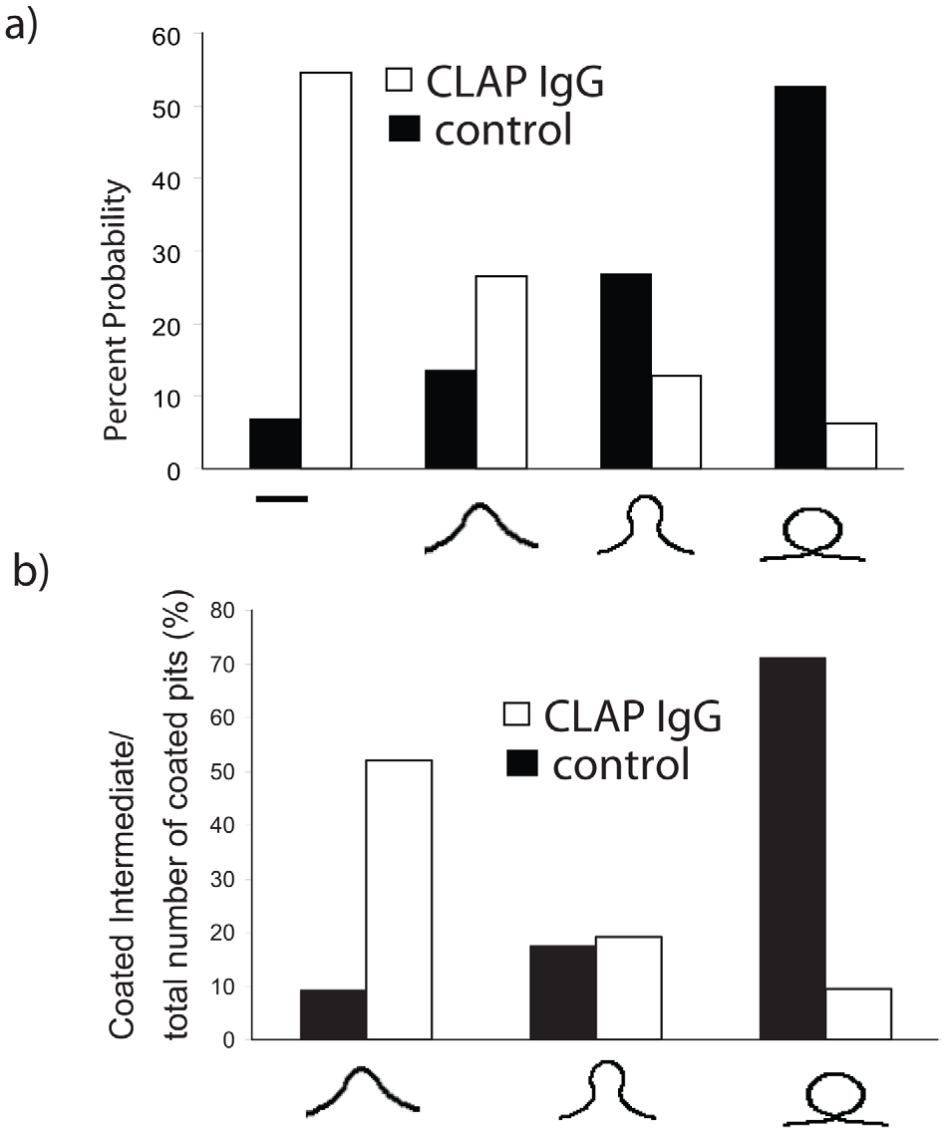
Distribution of vesicular intermediates. (a) Calculated and (b) experimental probability of observing a clathrin-coated vesicular bud of given size in WT cells (filled) and CLAP IgG injected cells (unfilled). In the calculated histogram, the four categories defined are based on the progression of bud growth. Category 2 includes all buds for which bud diameter is less compared to the neck radius, while category 4 includes all buds for which bud diameter is more compared to the neck radius. Category 3 is an intermediate case between category 2 and 4.

Recently, Jakobsson et al. [19] studied the role of epsin in synaptic vesicle endocytosis by inhibiting the interactions of epsin with clathrin using a CLAP antibody and those of epsin with PIP2 on membrane using an ENTH antibody. By microinjecting the CLAP antibody into neuronal cells, they observed that while the total extent of clathrin coated regions in the periactive zone on the plasma membrane remained the same, the observed fractions of the coated regions in different stages of coated-vesicle budding prior to scission were altered in a dramatic fashion, (see Fig. 5b): in the control wildtype (WT) cells, coated structures resembling a mature vesicular bud are more probable in comparison to planar structures and early intermediates; however, upon addition of CLAP, the early intermediates are stabilized and become more probable at the expense of the number of mature vesicular buds [19].

By computing E_c_ and E_r_ for different values of A_a_ in the capsid model, we determine the energetics of the clathrin coated vesicular bud E_t_ versus coat area, A_a_ for the capsid model (see Fig. S6). Number of epsins in WT (control) cell = 21: this number differs slightly from 23, the estimate for the epsin shell model, because R(s_0_) for the capsid model is slightly different from that for the shell model. We also computed probability of observing different coated-intermediates of vesicular structures as P∝exp(−E_t_(A_a_)/k_B_T) as depicted in Fig. 5a. The predicted distribution of vesicular intermediates (Fig. 5a) closely matches the experimental distribution reported by Jakobsson et al. [19] (see Fig. 5b). For modeling the clathrin-coated vesiculation in CLAP IgG injected cells, we compute the number of epsins as N_epsins_(CLAP cells) = N_epsins_(WT cells)*A_a_(vesicles in CLAP injected cells)/A_a_(in WT cells) = 33. The ratio of the respective areas ( = 1.6) is determined based on the experimental observations of increase in the size of the coated intermediates in CLAP injected cells relative to WT cells [19]. Remarkably, with N_epsins_ = 33 and ε_epsin_ = −14 k_B_T (reduced from −23 k_B_T due to the abrogation of the CLAP-clathrin/AP-2 interaction), we find not only that E_t_(A_a_) increases monotonically with A_a_ (a reversal in trend, see Fig. S6) but also the probability P∝exp(−E_t_(A_a_)/k_B_T) quantitatively matches the experimentally observed distribution in CLAP IgG injected cells, (compare Figs. 5a and 5b). We note that even though N_epsins_ increase in the CLAP IgG injected cells relative to wildtype, the size of the bud likely increases due to a lack of templating of epsins; arguably, there is lack of bond-orientational order as the CLAP domains of epsin can no-longer bind the periodic clathrin lattice. Corroborating this view, many extended coated structures (*cisternae*) also appear in the experiments with CLAP IgG injected cells [19]. Furthermore, according to the predictions of our model, disrupting the epsin-membrane interaction (i.e., by targeting the ENTH domain of epsin) completely abrogates E_r_ and should make the coated vesicular bud highly unfavorable. Indeed, consistent with this view, in cells microinjected with ENTH antibodies the extent of clathrin-coated structures decreased by over 90% [19]. Regarding the comparison in Fig. 5, we re-iterate that the fraction (or histogram) is proportional to exponential of the energy. Hence a small error in energy (of the order of k_B_T which is 0.6 kcal/mol at T = 300 K) can lead to a large change in the fraction [exp(0.6)≈factor of 2]. Hence, an order of magnitude agreement in histograms between theory and experiment in the trends of the intermediate shapes implies that the energetics agree even more closely.

## Discussion

In conclusion, we have presented a minimal mesoscale model which we believe imposes the correct spatial as well as thermodynamic constraints, and quantitatively explains several experimental observations on the process of vesicle nucleation induced by the clathrin-coated assembly prior to vesicle scission in CDE. We re-iterate that the input to our model is the membrane bending rigidity, spacing between epsins bound to the clathrin coat, and the curvature-field imposed by each bound epsin, which have all been determined using independent biophysical experiments. For these choices of input, our calculations then yield the membrane profiles for different sizes of the clathrin coat. Based on the number of shells of epsins accommodated on the clathrin coat (which depends on the size of the coat), and the circumference of each shell (which depends on the coat/membrane deformation), the number of epsins is calculated. Thus, the number of epsins, the membrane profile, and the deformation energy are outputs of our model. While our model does not include nucleation of the clathrin coat or scission of a mature coated vesicular-bud, our results identify a unique dual role for the tubulating protein epsin: multiple epsins localized spatially and orientationally collectively play the central role of a curvature inducing capsid; in addition, epsin serves the role as an adapter in binding the clathrin coat to the membrane. Our results also suggest an important role for the clathrin lattice, namely in the spatial- and orientational-templating of epsins for providing the appropriate curvature field for vesicle budding. We suggest that there exists a critical size (area) of the coat above which a vesicular bud with a constricted neck resembling a mature vesicle is stabilized. Based on the strong dependence of the vesicle diameter on the bending rigidity, we suggest that the variability in bending stiffness due variations in membrane composition with cell type can explain the experimentally observed variability on the size of clathrin-coated vesicles, which typically range 50–100 nm.

Apart from providing a mechanistic description of the budding process in CDE, our model provides estimates for the number of epsins involved in stabilizing a coated vesicle, and without any direct fitting, reproduces the experimentally observed shapes of vesicular intermediates as well as their probability distributions quantitatively in wildtype as well as CLAP IgG injected neuronal cell experiments. We consider such an agreement to be a strong validation for the basis of our model. These model predictions can further be tested by engineering mutations in epsin, clathrin, and AP-2 all of which are predicted to influence the distribution of coated structures. The framework of our approach is generalizable to vesicle nucleation in clathrin-independent endocytosis. Indeed, based on our results we can speculate that alternative mechanisms (such as receptor clustering) which can provide a hexatic bond-orientational templating of epsins on the membrane can facilitate vesicle-bud formation independent of CDE [11]. Future modeling work will address spatial distribution of curvature inducting proteins on vesicle nucleation [20].

## Supporting Information

Figure S1 The capsid model. Three different membrane deformation profiles under the influence of clathrin imposed curvature for s_0_ = 25, 50 and 70 nm. For s_0_ = 70 nm, membrane shape is reminiscent of a clathrin-coated vesicle. Inset (top): A schematic of the membrane profile explaining various symbols in the surface evolution methodology. The full membrane profile is obtained by rotating the curve by 2π about the z-axis. Inset (bottom) shows spontaneous curvature function experienced by the membrane due to the clathrin coat assembly in the capsid model.
(0.14 MB PDF)

Figure S2 The capsid model. Curvature deformation energy of the membrane versus the area of the clathrin coat, A_a_(s_0_) for different values of s_0_: 25nm–70nm. Inset: vesicle neck-radius R(s_0_) plotted against coat area A(s_0_) for different values of s_0_: 25 nm–70 nm.
(0.14 MB PDF)

Figure S3 Epsin shell model. Radius R versus s in the epsin shell model.
(0.17 MB PDF)

Figure S4 Epsin shell model. Determination of the range parameter b as a function of bending rigidity.
(0.05 MB PDF)

Figure S5 a) A schematic (corresponding to a mature bud in Fig. 3) showing membrane and three concentric shells of epsin present on the membrane. These shells of epsin are 18.5 nm (measured along the membrane arc-length, s) far from each other. Each shell of epsin imposes a intrinsic curvature onto the membrane. b) Epsin Shell Model- Comparison of curvature field functions in the epsin shell model (solid line) and the capsid model (dashed line).
(0.08 MB PDF)

Figure S6 Energetics of the clathrin coated vesicular bud E_t_ versus coat area, A_a_ for the capsid model.
(0.19 MB PDF)

Text S1 Membrane shape equations and details of the numerical scheme.
(0.05 MB PDF)

Text S2 The capsid model.
(0.03 MB PDF)
