# Supplementary material for: Minimal Mesoscale Model for Protein-Mediated Vesiculation in Clathrin-Dependent Endocytosis
Source: PLoS Comput Biol. 2010 Sep 9;6(9):e1000926. doi: 10.1371/journal.pcbi.1000926 (PMC2936510; doi:10.1371/journal.pcbi.1000926)
Supplement: Figure S1 — The capsid model. Three different membrane deformation profiles under the influence of clathrin imposed curvature for s0 = 25, 50 and 70 nm. For s0 = 70 nm, membrane shape is reminiscent of a clathrin-coated vesicle. Inset (top): A schematic of the membrane profile explaining various symbols in the surface evolution methodology. The full membrane profile is obtained by rotating the curve by 2π about the z-axis. Inset (bottom) shows spontaneous curvature function experienced by the membrane due to the clathrin coat assembly in the capsid model. (0.14 MB PDF) [file pcbi.1000926.s001.pdf]

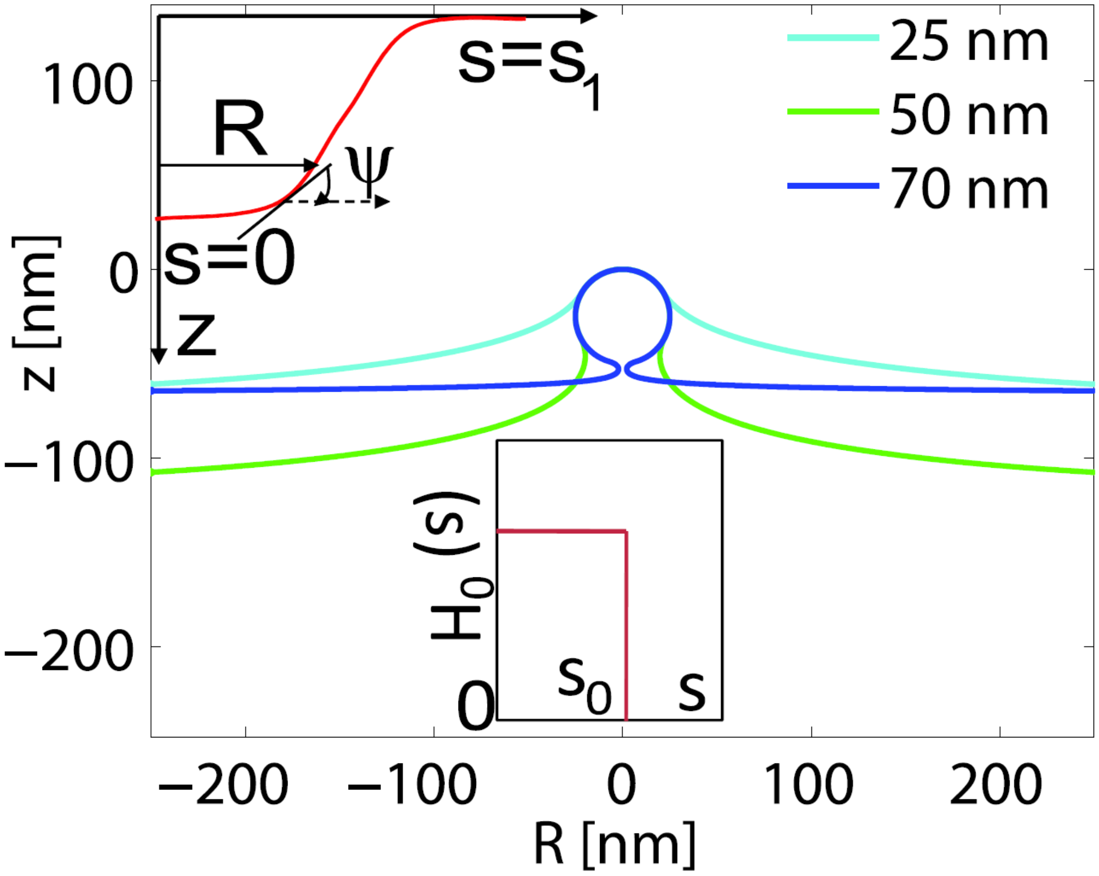

Figure S1: Capsid Model- Three different membrane deformation profiles under the influence of clathrin imposed curvature for s_0=25, 50 and 70 nm. For s_0=70 nm, membrane shape is reminiscent of a clathrin-coated vesicle. Inset (top): A schematic of the membrane profile explaining various symbols in the surface evolution methodology. The full membrane profile is obtained by rotating the curve by 2π about the z-axis. Inset (bottom) shows spontaneous curvature function experienced by the membrane due to the clathrin coat assembly in the capsid model.
