# Supplementary material for: Minimal Mesoscale Model for Protein-Mediated Vesiculation in Clathrin-Dependent Endocytosis
Source: PLoS Comput Biol. 2010 Sep 9;6(9):e1000926. doi: 10.1371/journal.pcbi.1000926 (PMC2936510; doi:10.1371/journal.pcbi.1000926)
Supplement: Figure S2 — The capsid model. Curvature deformation energy of the membrane versus the area of the clathrin coat, Aa(s0) for different values of s0: 25nm–70nm. Inset: vesicle neck-radius R(s0) plotted against coat area A(s0) for different values of s0: 25 nm–70 nm. (0.14 MB PDF) [file pcbi.1000926.s002.pdf]

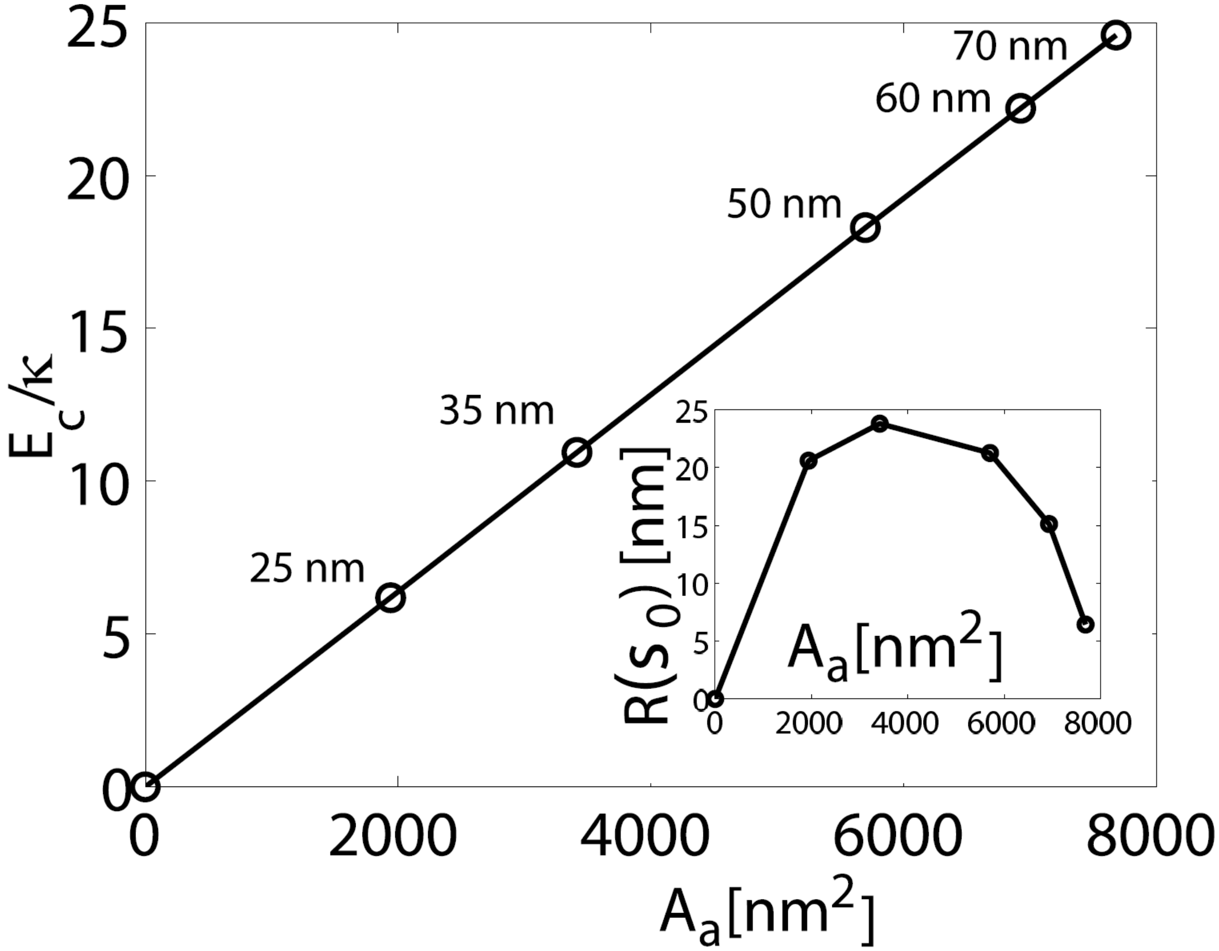

Figure S2: Capsid Model- Curvature deformation energy of the membrane versus the area of the clathrin coat, $A_a(s_0)$ for different values of s_0: 25nm-70nm. Inset: vesicle neck-radius $R(s_0)$ plotted against coat area $A(s_0)$ for different values of s_0: 25nm-70 nm.
