# Supplementary figures and images for: Minimal Mesoscale Model for Protein-Mediated Vesiculation in Clathrin-Dependent Endocytosis
Source: PLoS Comput Biol. 2010 Sep 9;6(9):e1000926. doi: 10.1371/journal.pcbi.1000926 (PMC2936510; doi:10.1371/journal.pcbi.1000926)

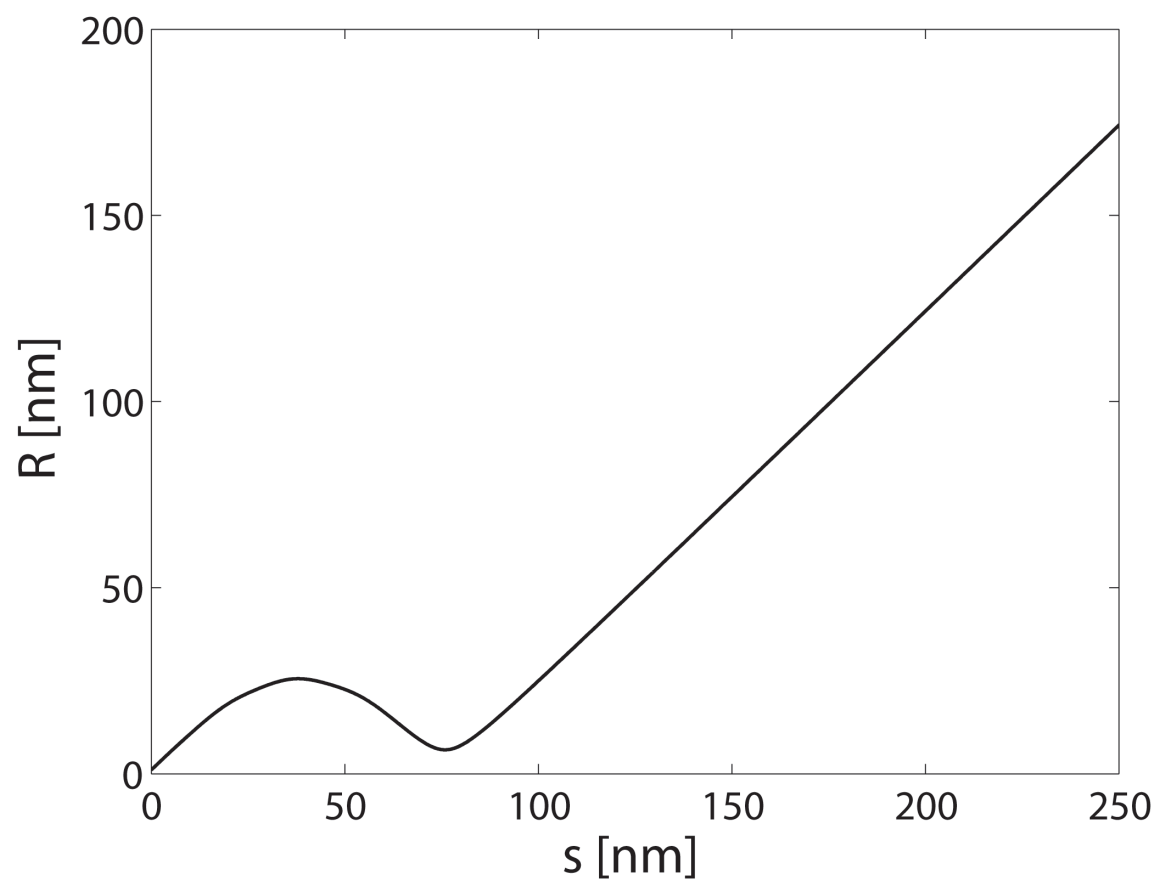

**Figure S3:** Epsin Shell Model- Radius  $R$  versus  $s$  in the epsin shell model.

Supplement: Figure S3 — Epsin shell model. Radius R versus s in the epsin shell model. (0.17 MB PDF) [file pcbi.1000926.s003.pdf]
