# Supplementary material for: Minimal Mesoscale Model for Protein-Mediated Vesiculation in Clathrin-Dependent Endocytosis
Source: PLoS Comput Biol. 2010 Sep 9;6(9):e1000926. doi: 10.1371/journal.pcbi.1000926 (PMC2936510; doi:10.1371/journal.pcbi.1000926)
Supplement: Figure S4 — Epsin shell model. Determination of the range parameter b as a function of bending rigidity. (0.05 MB PDF) [file pcbi.1000926.s004.pdf]

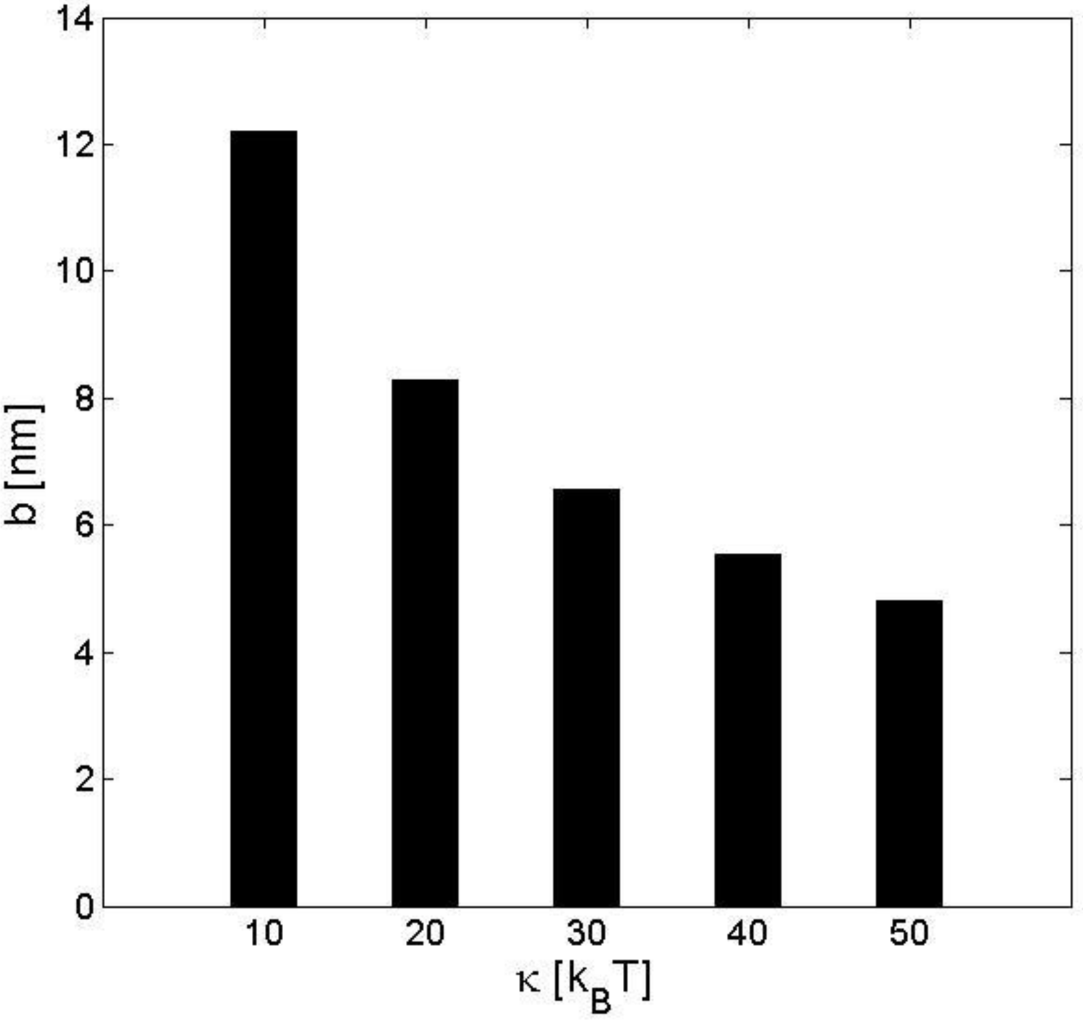

Figure S4: Epsin Shell Model- Determination of the range parameter b as a function of bending rigidity.
