# Supplementary material for: Minimal Mesoscale Model for Protein-Mediated Vesiculation in Clathrin-Dependent Endocytosis
Source: PLoS Comput Biol. 2010 Sep 9;6(9):e1000926. doi: 10.1371/journal.pcbi.1000926 (PMC2936510; doi:10.1371/journal.pcbi.1000926)
Supplement: Figure S5 — a) A schematic (corresponding to a mature bud in Fig. 3) showing membrane and three concentric shells of epsin present on the membrane. These shells of epsin are 18.5 nm (measured along the membrane arc-length, s) far from each other. Each shell of epsin imposes a intrinsic curvature onto the membrane. b) Epsin Shell Model- Comparison of curvature field functions in the epsin shell model (solid line) and the capsid model (dashed line). (0.08 MB PDF) [file pcbi.1000926.s005.pdf]

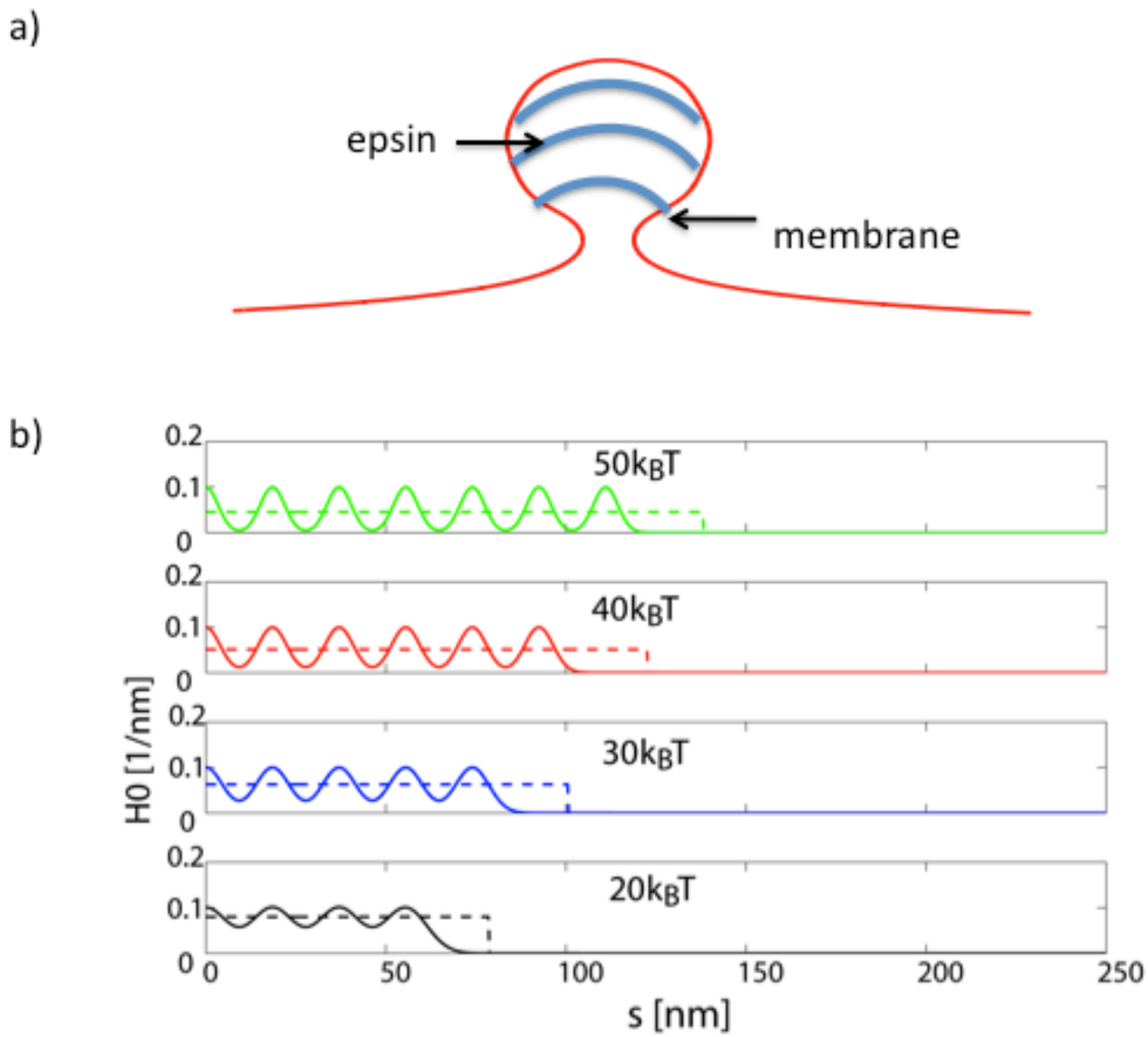

Figure S5: a) A schematic (corresponding to a mature bud in Fig. 3) showing membrane and three concentric shells of epsin present on the membrane. These shells of epsin are 18.5 nm (measured along the membrane arc-length, s) far from each other. Each shell of epsin imposes a intrinsic curvature onto the membrane b)Epsin Shell Model- Comparison of curvature field functions in the epsin shell model (solid line) and the capsid model (dashed line).
