# Supplementary material for: Minimal Mesoscale Model for Protein-Mediated Vesiculation in Clathrin-Dependent Endocytosis
Source: PLoS Comput Biol. 2010 Sep 9;6(9):e1000926. doi: 10.1371/journal.pcbi.1000926 (PMC2936510; doi:10.1371/journal.pcbi.1000926)
Supplement: Figure S6 — Energetics of the clathrin coated vesicular bud Et versus coat area, Aa for the capsid model. (0.19 MB PDF) [file pcbi.1000926.s006.pdf]

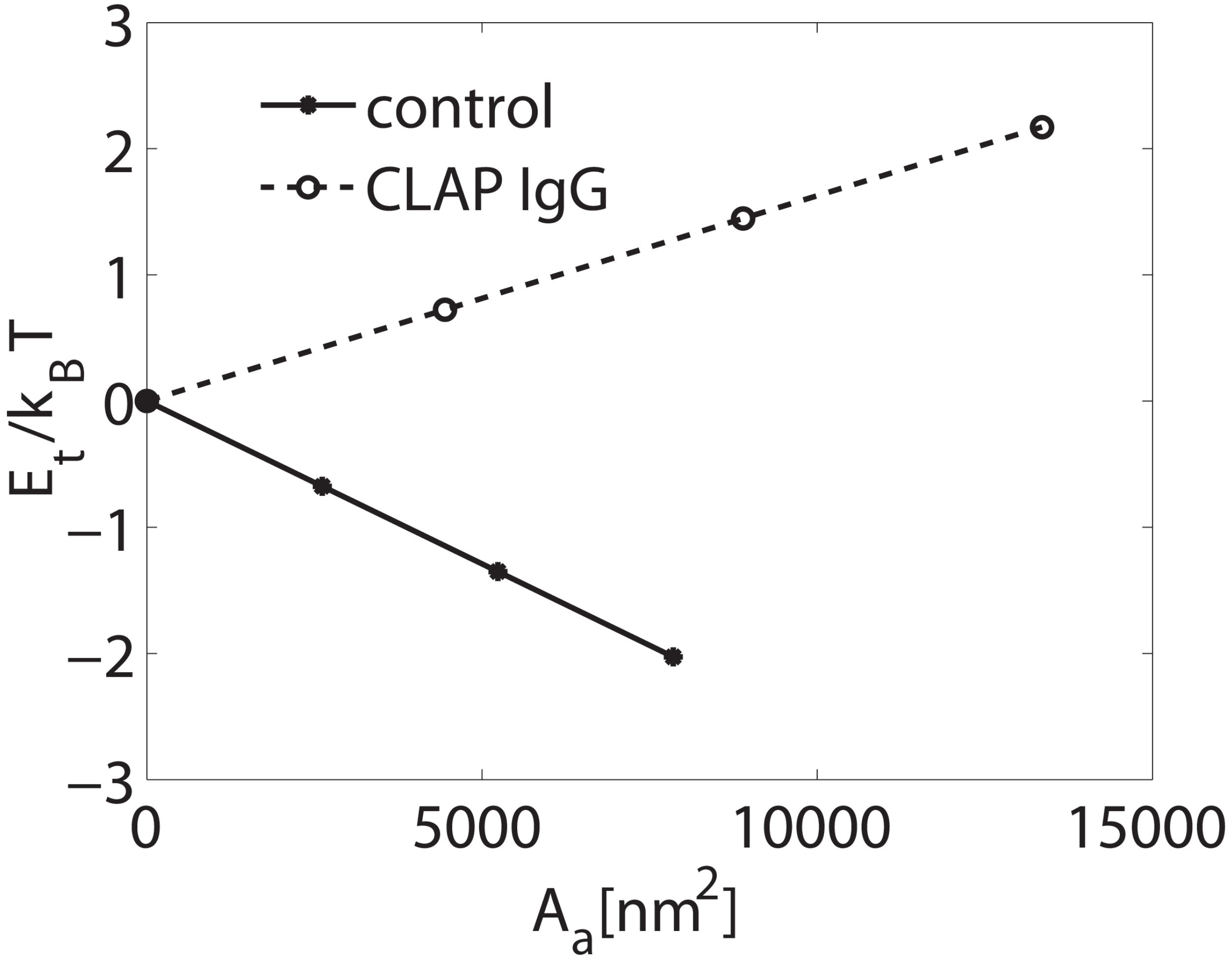

Fig. S6. Energetics of the clathrin coated vesicular bud E_t versus coat area, A_a for the capsid model.
