# Supplementary material for: Minimal Mesoscale Model for Protein-Mediated Vesiculation in Clathrin-Dependent Endocytosis
Source: PLoS Comput Biol. 2010 Sep 9;6(9):e1000926. doi: 10.1371/journal.pcbi.1000926 (PMC2936510; doi:10.1371/journal.pcbi.1000926)
Supplement: Text S1 — Membrane shape equations and details of the numerical scheme. (0.05 MB PDF) [file pcbi.1000926.s007.pdf]

Text S1: Membrane Energy Minimization

Assuming axial symmetry, we introduce a surface of revolution approach to model the membrane at equilibrium. We consider a generating curve γ parameterized by arc length s lying in the $x - z$ plane. The curve γ is expressed as

$$\gamma(0, s_1) \rightarrow \mathbf{R}^3 \gamma(s) = (R(s), 0, z(s)) \tag{S1.1}$$

where s_1 is the total arc-length. This generating curve leads to a global parametrization of the membrane expressed as

$$X : (0, s_1) \times (0, 2\pi) \rightarrow \mathbf{R}^3 \tag{S1.2}$$
$$X(s, u) = (R(s)cos(u), R(s)sin(u), z(s)) \tag{S1.3}$$

where u is the angle of rotation about z-axis. With this parametrization, the mean curvature H and the Gaussian curvature K are given as follows respectively

$$H = -\frac{z' + R(z'R'' - z''R')}{R} \tag{S1.4}$$
$$K = -\frac{R''}{R} \tag{S1.5}$$

where the prime indicates differential with respect to arc-length s. The expressions obtained above for the mean curvature and the gaussian curvature are quite complicated. To simplify them, an extra variable ψ where $\psi(s)$ is the angle between the tangent to the curve and the horizontal direction, is introduced. The declation of this extra variable introduces following two geometric constraints:

$$R' = cos(\psi(s)) \tag{S1.6}$$
$$z' = -sin(\psi(s)) \tag{S1.7}$$

These two constraints lead to the following simplified expressions for the mean curvature and the gaussian curvature.

$$H = \psi' + \frac{sin(\psi(s))}{R(s)} \tag{S1.8}$$
$$K = \psi' \frac{sin(\psi(s))}{R(s)} \tag{S1.9}$$

The membrane free energy E is defined by

$$E = \int_0^{2\pi} \int_0^{s_1} \frac{\kappa}{2}[H - H_o]^2 + \overline{\kappa}K + \sigma dA \tag{S1.10}$$

where dA is the area element given by $Rdsdu$, κ is the bending rigidity, $\overline{\kappa}$ is the splay modulus, σ is the line tension. Substituting for H, K, we obtain the following expression for the free energy

$$E = \int_0^{2\pi} \int_0^{s_1} (\frac{\kappa}{2}[\psi' + \frac{sin(\psi(s))}{R(s)} - H_o]^2 + \overline{\kappa}\psi' \frac{sin(\psi(s))}{R(s)} + \sigma)Rdsdu \tag{S1.11}$$

We now proceed to determine the minimum-energy shape of the membrane. The condition that specifies the the minimum-energy profile is that, the first variation of the energy should be zero. That is

$$\delta E = 0 \tag{S1.12}$$

subject to the geometric constraints $R' = cos(\psi(s)), z' = -sin(\psi(s))$. These constraints can be reexpressed in an integral form as follows

$$\int_0^{s_1} R' - cos(\psi(s))ds = 0 \tag{S1.13}$$

$$\int_0^{s_1} z' + sin(\psi(s))ds = 0 \tag{S1.14}$$

Introducing lagrange multipliers, we solve our constrained optimization problem as follows. We introduce the Lagrange function ν, η and minimize the quantity F

$$F = \int_0^{2\pi} \int_0^{s_1} \left(\frac{\kappa}{2}[\psi' + \frac{sin(\psi(s))}{R(s)} - H_o]^2 + \overline{\kappa}\psi' \frac{sin(\psi(s))}{R(s)} \sigma \right) R ds du + \nu \int_0^{s_1} R' - cos(\psi(s))ds + \eta \int_0^{s_1} z' + sin(\psi(s))ds \tag{S1.15}$$

Since the integrand of the double integral is independent of u, F simplifies to

$$F = 2\pi \int_0^{s_1} \left\{ \frac{\kappa R}{2} \left[\psi' + \frac{sin(\psi(s))}{R(s)} - H_o \right]^2 + \overline{\kappa}\psi' sin(\psi(s)) + \sigma R + \nu \left[R' - cos(\psi(s)) \right] + \eta \left[z' + sin(\psi(s)) \right] \right\} ds \tag{S1.16}$$

The minimization problem is then expressed as

$$\delta F = 0 \tag{S1.17}$$

We denote the integrand of functional in Eq. S1.16 as L.

$$L = \frac{\kappa R}{2} \left[\psi' + \frac{sin(\psi(s))}{R(s)} - H_o \right]^2 + \overline{\kappa}\psi' sin(\psi(s)) + \sigma R + \nu \left[R' - cos(\psi(s)) \right] + \eta \left[z' + sin(\psi(s)) \right] \tag{S1.18}$$

So, we get

$$F = 2\pi \int_o^{s_1} L ds \tag{S1.19}$$

We interpret F in as a functional of the variables $s_1, R, z, \psi, \eta, \nu$. We denote variables R, z, ψ, η, ν by p_i. Now the "generalized" or (non-simultaneous) variation ΔF is expressed as

$$\Delta F = 2\pi\Delta \int_0^{s_1} L(s, p_i) ds \tag{S1.20}$$

For a detailed description of terminology used and the following method, readers are referred to [1]. Performing the generalized variation, we get

$$\Delta F = \int_0^{s_1} \left(\frac{\partial L}{\partial p_i} - \frac{d}{ds}\frac{\partial L}{\partial p_i'} \right) \delta p_i ds + \left[\frac{\partial L}{\partial p_i'} \Delta p_i \right]_0^{s_1} + \left[\left(L - \frac{\partial L}{\partial p_i'} p_i' \right) \Delta s \right]_0^{s_1} \tag{S1.21}$$

At equlibrium, the integral in Eq. S1.21 should be zero, which leads to following Euler-Lagrange eqns.

$$\frac{\partial L}{\partial p_i} - \frac{d}{ds}\frac{\partial L}{\partial p_i'} = 0 \tag{S1.22}$$

Therefore, the boundary conditions at s_1 are specified by the relationship

$$\left[\frac{\partial L}{\partial p_i'} \Delta p_i \right]_0^{s_1} + \left[\left(L - \frac{\partial L}{\partial p_i'} p_i' \right) \Delta s \right]_0^{s_1} = 0 \tag{S1.23}$$

To simplify the boundary conditions, we define a new function H (analogous to Hamiltonian) which is of the form

$$H = -L + p'_i \frac{\partial L}{\partial p'_i} \tag{S1.24}$$

Now, the boundary term simplifies to

$$[-H\Delta s]_0^{s_1} + \left[\frac{\partial L}{\partial p'_i}\Delta p_i\right]_0^{s_1} = 0 \tag{S1.25}$$

The above two key equations results in the following series of equations that describe the membrane equilibrium profile.

$$\frac{\partial L}{\partial \psi} - \frac{d}{ds}\frac{\partial L}{\partial \psi'} = 0 \tag{S1.26}$$
$$\frac{\partial L}{\partial R} - \frac{d}{ds}\frac{\partial L}{\partial R'} = 0 \tag{S1.27}$$
$$\frac{\partial L}{\partial z} - \frac{d}{ds}\frac{\partial L}{\partial z'} = 0 \tag{S1.28}$$
$$\frac{\partial L}{\partial \nu} = 0 \tag{S1.29}$$
$$\frac{\partial L}{\partial \eta} = 0 \tag{S1.30}$$
$$[-H\Delta s]_0^{s_1} = 0 \tag{S1.31}$$
$$\left[\frac{\partial L}{\partial \psi'}\Delta \psi\right]_0^{s_1} = 0 \tag{S1.32}$$
$$\left[\frac{\partial L}{\partial R'}\Delta R\right]_0^{s_1} = 0 \tag{S1.33}$$
$$\left[\frac{\partial L}{\partial z'}\Delta z\right]_0^{s_1} = 0 \tag{S1.34}$$
$$\left[\frac{\partial L}{\partial \nu'}\Delta \nu\right]_0^{s_1} = 0 \tag{S1.35}$$
$$\left[\frac{\partial L}{\partial \eta'}\Delta \eta\right]_0^{s_1} = 0 \tag{S1.36}$$

Recall that

$$L = \frac{\kappa R}{2}\left[\psi' + \frac{sin(\psi(s))}{R(s)} - H_o\right]^2 + \overline{\kappa}\psi' sin(\psi(s)) + \sigma R + \nu(R' - cos(\psi(s)) + \eta(z' + sin(\psi(s)) \tag{S1.37}$$

We now take the spontaneous curvature $H_o = \phi(s)$ where $\phi(s)$ is an appropriately chosen function. The lagrangian L becomes

$$L = \frac{\kappa R}{2}\left[\psi' + \frac{sin(\psi(s))}{R(s)} - \phi(s)\right]^2 + \overline{\kappa}\psi' sin(\psi(s)) + \sigma R + \nu(R' - cos(\psi(s)) + \eta(z' + sin(\psi(s)) \tag{S1.38}$$

The Lagrangian, L depends on the arc-length s due to the (in general) spatially-varying spontaneous curvature, $\phi(s)$. Hence the Hamiltonian, H is not a conserved quantity along s. This is in contrast to the conserved Hamiltonian in [2] and [3] since those authors assumed a constant spontaneous curvature along the membrane.

Since for a topologically-invariant transformation, the contribution to gaussian curvature to functional F is constant, we do not expect to see any terms involving $\overline{\kappa}$ in following expressions. The Eq. S1.26 results in the following

expression

$$\psi'' = \frac{cos(\psi)sin(\psi)}{R^2} - \frac{\psi' cos(\psi)}{R} + \frac{\nu sin(\psi)}{R\kappa} + \frac{\eta cos(\psi)}{R\kappa} + \phi'(s) \tag{S1.39}$$

Note that in the above expression, we retain the $\phi'(s)$ term since, in general, spontaneous curvature can be a function of arc-length, s.

The Eq. S1.27 gives the following expression for ν'

$$\nu' = \frac{\kappa[\psi' - \phi(s)]^2}{2} - \frac{\kappa sin^2(\psi)}{2R^2} + \sigma \tag{S1.40}$$

The Eq. S1.28 gives the following expression for η

$$\eta' = 0 \tag{S1.41}$$

The Eq. S1.29 gives the following expression

$$R' = cos(\psi(s)) \tag{S1.42}$$

The Eq. S1.30 gives the following expression

$$z' = -sin(\psi(s)) \tag{S1.43}$$

Since, s is fixed when $s = 0$, $\Delta s = 0$ when $s = 0$. Hence, Eq. S1.31 reduces to

$$[-H\Delta s]_{s_1} = 0 \tag{S1.44}$$

Since, $\Delta s \neq 0$ when $s = s_1$, we conclude that at $s = s_1$, $H = 0$. Deriving H from L, we get

$$H = \kappa \frac{R}{2}\left[\psi'^2 - \left(\frac{sin\psi}{R} - \phi\right)^2\right] - \sigma R + \nu cos\psi - \eta sin\psi = 0 \tag{S1.45}$$

A similar result was obtained by Seifert [3] where they showed that when the total arc-length s_1 is not known a priori (i.e. s_1 is free) the Hamiltonian, $H(s_1) = 0$.

From Eq. S1.32, we get

$$\kappa\left[R\left(\psi' + \frac{sin\psi}{R} - \phi\right)\Delta\psi\right]_0^{s_1} = 0 \tag{S1.46}$$

From Eq. S1.33, we get

$$[\nu\Delta R]_0^{s_1} = 0 \tag{S1.47}$$

From Eq. S1.34, we get

$$[\eta\Delta z]_0^{s_1} = 0 \tag{S1.48}$$

There are no terms involving ν' and η' in definition of L in S1.38. Hence, Eq. S1.35 and S1.36 does not provide any information. Since we have second order ODE for ψ, first order ODE for R, z, ν, η and since s_1 is also unknown, in total we need 7 boundary conditions. Equation S1.45 provides us with 1 equation. We still need to provide 6 additional equations. For clarity, s is zero when the curve has zero radius.

We consider few example cases for the boundary conditions:

S1.1 Case I

At $s = 0$, let's specify $\psi = 0$, $R = 0$ and $z = 0$. So, at $s = 0$, $\Delta\psi$, ΔR, Δz are all zero. Since, we have not specified ψ, R, z at $s = s_1$, we have $\Delta\psi$, ΔR, Δz are all non-zero at $s = s_1$. Use of Eq. S1.46, S1.47 and S1.48 tells us that at

$$R(s_1)\left(\psi'(s_1) + \frac{sin\psi(s_1)}{R(s_1)} - \phi(s_1)\right) = 0 \quad \text{(S1.49)}$$

$$\nu(s_1) = 0 \quad \text{(S1.50)}$$

$$\eta(s_1) = 0 \quad \text{(S1.51)}$$

If we assume in S1.49 that $R(s_1) \neq 0$, then we have

$$\left(\psi'(s_1) + \frac{sin\psi(s_1)}{R(s_1)} - \phi(s_1)\right) = 0 \quad \text{(S1.52)}$$

Substitution of this relation into Eq. S1.45 along with using Eq. S1.50 and S1.51 results into $R(s_1) = 0$ which invalidates our assumption. So, R has to be zero at s_1, i.e. the curve has both its ends at the z-axis and so it looks like a sphere. Hence, this boundary condition is not applicable for the pinned membrane.

S1.2 Case II

At $s = 0$, let's specify $\psi = 0$, $R = 0$ and $z = 0$ and at $s = s_1$, we specify $\psi = 0$, $R = R_0$ and $z = z_0$. With these conditions, Eq. S1.45 reduces to

$$\nu(s_1) = \sigma R_0; \quad \text{(S1.53)}$$

S1.3 S2 Numerical Algorithm

S1.3.1 Analytical Solution for initial guess

When $\sigma = 0$, we expect the solution to be $H = \phi$, $R' = cos\psi$ and $\nu = 0$. In this section, we show that above three equations are indeed a solution to the Eq. S1.39, S1.40 and S1.42 when $\sigma = 0$. Now, $H = \phi$ tells us that

$$\psi' + \frac{sin\psi}{R} = \phi \quad \text{(S1.54)}$$

We differentiate S1.54 w.r.to s and use $R' = cos\psi$ to get

$$\psi'' = -\frac{\psi' cos(\psi)}{R} + \frac{cos\psi sin\psi}{R^2} + \phi' \quad \text{(S1.55)}$$

Now, above equation along with $\nu = 0$ satisfies eq. S1.39. Substituting, $\nu = 0$ and $\psi' = -\frac{sin\psi}{R} + \phi$ in S1.40, we get

$$0 = \frac{\kappa}{2}\left[\left(\frac{sin\psi}{R}\right)^2 - \left(\frac{sin\psi}{R}\right)^2\right] \quad \text{(S1.56)}$$

This proves that when $\sigma = 0$, $H = \phi$, $R' = cos\psi$ and $\nu = 0$ are the solution. Note we assume $\nu = 0$ so that it also satisfies the boundary condition, i.e. $\nu(s_1) = 0$. This analytical solution might provides us with a very good initial guess when $\sigma \neq 0$. However, since $H = \phi$ is a first order differential equation, it satisfies only one boundary condition. Hence, in general, solution of $H = \phi$ will not satisfy both boundary conditions for ψ. Hence, in general, $H = \phi$ is not a solution for our system. $H = \phi$ satisfies both boundary conditions iff $\int_0^{s_1} \psi' ds = 0$, i.e. $\int_0^{s_1} \frac{sin\psi}{R} - \phi ds = 0$. So, we rather use a different approach to calculate the initial guess: We know that for ψ to satisfy both the boundary conditions, $\int_0^{s_1} \psi' ds$ has to be zero. When $\int_0^{s_1} \phi ds = 0$, then, we know that $\psi' = \phi$ provides us with a good intial guess consistent with the boundary conditions. When $\int_0^{s_1} \phi ds \neq 0$, we define $\epsilon = \int_0^{s_1} \phi ds$. Then we know that

$\int_0^{s_1} (\phi - \epsilon/s_1)\, ds = 0$. Now, we define our initial guess to be $\psi' = \phi - \epsilon/s_1$. Integrating this expression, we get $\psi = \int_0^s \phi ds - \epsilon s/s_1$ as our initial guess.

S1.3.2 Numerical Solution

We specify guess value for s_1 and then calculate the guess value for ψ using the method outlined in section S1.3.1. Once initial value of ψ is available, we also calculate initial value of R and ν using Eq. S1.42 and Eq. S1.40 respectively. Then we solve the Eq. S1.39, S1.40, S1.42 and S1.43 along with the boundary conditions specified in section S1.2 and Eq. S1.53 numerically. From the results of these calculations, we calculate $R(s_1)$. Convergence of $R(s_1)$ to R_0 within some tolerance by varying s_1 indicates the converged membrane profile.

References

[1] Vujanovic, B. D. and Atanackovic, T. M. (2004) An introduction to modern variational techniques in mechanics and engineering (Springer), pp. 132.

[2] M. Deserno, Phys Rev E 69, 031903 (2004).

[3] U. Seifert, K. Berndl, and R. Lipowsky, Phys Rev A 44, 1182 (1991).
