# Supplementary material for: Minimal Mesoscale Model for Protein-Mediated Vesiculation in Clathrin-Dependent Endocytosis
Source: PLoS Comput Biol. 2010 Sep 9;6(9):e1000926. doi: 10.1371/journal.pcbi.1000926 (PMC2936510; doi:10.1371/journal.pcbi.1000926)
Supplement: Text S2 — The capsid model. (0.03 MB PDF) [file pcbi.1000926.s008.pdf]

Text S2: The Capsid Model

In order to account for the effect of clathrin coat size on membrane deformation, we further simplify our model for curvature induction and assume that the clathrin-coat assembly acts as a capsid imposing a constant and radially symmetric mean radius of curvature field on the membrane with $H_0 = 0.08\ nm^{-1}$ [1]. This value is consistent with the typical clathrin-coated spherical vesicles imaged in neuronal cells [1]; thus, we set $H_0(s) = 0.08\ nm^{-1}$ if $s<s_0$ and $H_0(s) = 0$ if $s \geq s_0$, s_0 is the length of the clathrin coat assembly.
The close agreement between the membrane profiles obtained using the capsid model and the epsin shell model is evident from comparing Fig. S1 and Fig. 3. Our results also make clear that it is the embedded epsins on the clathrin coat that provide a major contribution to H_0. Fig. S2 depicts the energy of membrane deformation and the neck radius as a function of coat size for the capsid model. The energy of deformation of the fully mature bud is found to be $\approx 25\kappa$.

1. Chiu SW, Jakobsson E, Mashl RJ, Scott HL (2002) Cholesterol-induced modifications in lipid bilayers: a simulation study. Biophys J 83(4): 1842-1853.
